# Prevalence of Feline Coronavirus Shedding in German Catteries and Associated Risk Factors

**DOI:** 10.3390/v12091000

**Published:** 2020-09-08

**Authors:** Ute Klein-Richers, Katrin Hartmann, Regina Hofmann-Lehmann, Stefan Unterer, Michèle Bergmann, Anna Rieger, Christian Leutenegger, Nikola Pantchev, Jörg Balzer, Sandra Felten

**Affiliations:** 1 Clinic of Small Animal Medicine, Centre for Clinical Veterinary Medicine, LMU Munich, Veterinaerstrasse 13, 80539 Munich, Germany; hartmann@medizinische-kleintierklinik.de (K.H.); s.unterer@medizinische-kleintierklinik.de (S.U.); n.bergmann@medizinische-kleintierklinik.de (M.B.); riegeranna_lmu@web.de (A.R.); s.felten@medizinische-kleintierklinik.de (S.F.); 2 Clinical Laboratory, Department of Clinical Diagnostics and Services, Vetsuisse Faculty, University of Zurich, Winterthurerstrasse 260, 8057 Zurich, Switzerland; rhofmann@vetclinics.uzh.ch; 3 IDEXX Laboratories Inc., 2825 KOVR Drive, West Sacramento, CA 95605, USA; christian.leutenegger@antechmail.com; 4 IDEXX Laboratories, Humboldstr. 2, 70806 Kornwestheim, Germany; nikola-pantchev@idexx.com (N.P.); joerg-balzer@idexx.com (J.B.)

**Keywords:** feline coronavirus (FCoV), infection, real-time reverse transcriptase polymerase chain reaction (RT-qPCR), fecal samples, virus shedding, hygiene management, multi-cat household, feline infectious peritonitis (FIP)

## Abstract

The aim of this prospective study was to determine prevalence and potential risk factors of feline coronavirus (FCoV) shedding. Four consecutive fecal samples of 179 cats from 37 German breeding catteries were analyzed for FCoV ribonucleic acid (RNA) by real-time reverse transcriptase polymerase chain reaction (RT-qPCR). Prevalence of shedding was calculated using different numbers of fecal samples per cat (1–4) and different sampling intervals (5–28 days). Information on potential risk factors for FCoV shedding was obtained by a questionnaire. Risk factor analysis was performed using a generalized linear mixed model (GLMM). Most cats (137/179, 76.5%, 95% confidence interval (CI) 69.8–82.2) shed FCoV at least at once. None of the tested 37 catteries was free of FCoV. Prevalence calculated including all four (76.5%, 95% CI 69.8–82.2) or the last three (73.7%, 95% CI 66.8–79.7) samples per cat was significantly higher than the prevalence calculated with only the last sample (61.5%, 95% CI 54.2–68.3; *p* = 0.0029 and 0.0175, respectively). Young age was significantly associated with FCoV shedding while the other factors were not. For identification of FCoV shedders in multi-cat households, at least three fecal samples per cat should be analyzed. Young age is the most important risk factor for FCoV shedding.

## 1. Introduction

Feline coronaviruses (FCoV) are single-stranded, positive-sense ribonucleic acid (RNA) viruses of the family *Coronaviridae* [1,2], that exist as two pathotypes. Cats become infected with the avirulent pathotype, which usually causes no clinical signs or only mild enteritis. However, in up to 12% of the infected cats, a highly virulent mutant of FCoV will lead to the fatal syndrome of feline infectious peritonitis (FIP) [3,4,5]. FCoV is ubiquitous in most multi-cat environments, and it is important to detect FCoV shedders in these situations [6,7,8,9].

The prevalence of FCoV shedding has been investigated in several countries by testing fecal samples or rectal swabs for FCoV RNA by reverse transcriptase polymerase chain reaction (RT-PCR), and the results range from 31.8% to 100.0% [10,11,12,13,14,15,16,17,18,19,20,21,22,23]. Crowded living conditions and sharing litter boxes have been discussed as predisposing factors, but there are only a limited number of studies prospectively evaluating risk factors for FCoV shedding.

As of today, preventing FCoV infection is the only method of preventing FIP. Once a cat is infected, development of the fatal disease cannot be prevented. An inherited susceptibility to FIP has been shown in pedigree cats [24] but attempts to selectively breed resistant cats have failed [25]. Variants of the feline interferon-gamma gene (*fIFNG*) are thought to be associated with the risk of disease, but a study investigating the clinical use of this association to select cats for breeding could not show reliable results [26]. Another study evaluated the use of a novel feline infectious peritonitis virus (FIPV)-targeted RT-PCR to distinguish the avirulent pathotype from the virulent mutant, but the differentiation was not accurate [27].

FCoV-infected cats can shed the virus persistently, intermittently, or not at all [7,8,28,29]. Thus, for detection of FCoV shedders in multi-cat households, testing of several fecal samples has been recommended [7,8,9,30,31,32]. The optimal time interval between sampling, however, has not been determined prospectively [7,30,31,32].

The current prevalence of FCoV shedding in catteries in Germany is unknown, as are factors influencing this prevalence. Therefore, the aim of this study was to determine the prevalence of FCoV shedding in German breeding catteries and to evaluate associated risk factors. Additionally, serial fecal sampling at different time intervals was compared to single sampling in terms of efficacy to detect FCoV shedders within a multi-cat environment.

## 2. Materials and Methods

The prospective study included 179 cats from 37 catteries from all over Germany. Catteries were defined as private breeding establishments with at least one intact female cat and were included if they kept five or more cats. The study protocol was approved by the responsible veterinary authority (reference number 55.2-1-54-2532.2-14-2013). Owners gave their informed consent prior to participation.

Breeders were contacted via phone, email, or personally at cat shows. Those who were willing to participate were instructed to collect four consecutive fecal samples from an unlimited number of cats in their catteries; the samples were to be taken at intervals of five to 28 days. The first three samples were stored at −18 °C until all four samples were collected. Following collection of the fourth sample, which was kept unfrozen, all samples were immediately shipped refrigerated to the investigators. All four samples of each cat were analyzed for FCoV by real-time RT-PCR (RT-qPCR) using forward primer, reverse primer and probe as described previously [33]. Total nucleic acid was extracted using the MagVet™ Universal Purification Kit (ThermoFisher Scientific, Darmstadt, Germany) on an automated platform (KingFisher Flex 96; ThermoFisher Scientific, Darmstadt, Germany) according to the manufacturer’s instructions. RT-qPCR was performed using the LightCycler 480 system (Roche, Mannheim, Germany). The target gene was *FCoV 7b* gene (DQ010921.1). RT-qPCR was run with six quality controls, including RT-qPCR-positive controls (synthetic desoxyribonucleic acid (DNA) covering the RT-qPCR target region), RT-qPCR-negative controls (PCR-grade nuclease-free water), negative extraction controls (extraction positions filled with lysis solution and PCR-grade nuclease free water only), an internal positive control spiked into the lysis solution to monitor the nucleic acid extraction efficiency, and presence or absence of inhibitory substances (using lambda phage DNA), RNA pre-analytical quality control targeting feline ssr rRNA (18s rRNA) gene complex, and a swab-based environmental contamination monitoring control [12,33,34]. Samples with a Cp value below 40 were considered positive.

Overall prevalence of FCoV shedding was defined as the proportion of cats that tested positive for FCoV in at least one of the four samples. In order to evaluate if the number of analyzed samples per cat had a significant influence on the prevalence, prevalence was also calculated for one, two, or three samples per cat. This analysis always included the last samples of each cat; for example, sample number 4 was used for analysis of one sample per cat and samples number 3 and 4 were used for analysis of two samples per cat. Comparison of prevalence was performed using Fisher’s exact test.

The time intervals between the collection of each individual fecal sample ranged from five to 28 days, and each cat was assigned to one of four groups according to the longest interval between their four samplings ((group 1): longest interval 5–9 days; (group 2): longest interval 10–14 days; (group 3): longest interval 15–21 days; (group 4): longest interval 22–28 days). Prevalence was calculated separately for each of these groups and compared using Fisher’s exact test.

Evaluated risk factors included signalment (breed, gender, age, reproductive status) and anamnestic data (number of partner cats, hygiene management, outdoor access, feeding routine). Cat owners were asked to fill in a questionnaire (provided as Supplementary Material) for each cat including age, gender, breed, data on hygiene management (contact with cats from other households, litter box cleaning, disinfection routine), and general husbandry conditions (number of cats in the household, outdoor access, feeding, available space in general and per cat).

Univariate analysis was carried out using Fisher’s exact test for categorical variables and Mann–Whitney U test for continuous variables. A multivariate analysis was performed using statistical software (R Foundation for Statistical Computing, Vienna, Austria, Version 3.4.4), and in order to control for multiple observations of individual breeders, a generalized linear mixed model (GLMM) with logit link function and a random intercept per breeder was selected. This served as “breeder or cattery effect” to capture the impact of yet unknown risk factors for FCoV infection not considered in the questionnaire, such as environment ventilation, feeding interval, use of different disinfection agents and other management differences. Selection of variables was done with GLMM Lasso (R package glmmLasso, Andreas Groll (2016)). With Lasso, some variable coefficients are eliminated by the variable selection process in order to achieve a simple model containing only relevant risk factors. The optimal shrinking parameter was determined using Akaike information criterion (AIC).

## 3. Results

### 3.1. Prevalence of FCoV Shedding

None of the 37 tested catteries was entirely free of FCoV. The number of cats in the individual catteries ranged from five to 29 with a median number of 12 cats per cattery. The majority of catteries (23/37, 62.2%) kept more than ten cats in the household. The proportion of cats shedding FCoV within the individual catteries ranged from 12.5% to 100.0% of the sampled cats. Of 179 tested cats, 137 (76.5%, 95% confidence interval (CI) 69.8–82.2) tested positive for FCoV RNA in at least one of the four samples (Table 1). Prevalence calculated with different numbers of samples per cat are shown in Table 2. Prevalence was significantly lower when calculated from only one sample per cat compared to three or four samples per cat (*p* = 0.0175 and 0.0029, respectively). There was no significant difference when comparing prevalence calculated with different sampling intervals (Table 3).

### 3.2. Risk Factors and Breeders’ Effect

Univariate analysis suggested that breed and the number of cats in the household had a significant influence on the prevalence of FCoV shedding. However, multivariate analysis (GLMM), which captured the breeders’ influence, determined that the age of the cats was the only parameter significantly and independently associated with FCoV shedding. Cats under one year of age had a 2.5-times higher risk of shedding FCoV than cats between one and five years of age (*p* = 0.042, Odds Ratio (OR) 2.48, 95% CI 1.03–5.95). The number of cats per cattery, breed, hygiene management, husbandry conditions and outdoor access were not significantly associated with FCoV shedding in this population (Table 4 and Table 5). Outdoor access in this population referred to a fenced enclosure on the owner’s property, preventing cats from leaving the premises.

## 4. Discussion

Overall prevalence of FCoV shedding in 37 breeding catteries with more than five cats was 76.5% (95% CI 69.8–82.2). Other studies investigating FCoV shedding prevalence showed varying results depending on the examined cat population. In Canada, 86 of 185 (46.5%) healthy cats from shelters and private households were positive for FCoV RNA in the feces [16]. In Florida, USA, prevalence of FCoV shedding among cats entering an animal shelter was 58.0% in cats with diarrhea and 36.0% in cats with normal feces [19]. In California, USA, the overall prevalence of shedding upon admission to a shelter was 33%, with a prevalence of FCoV shedding in kittens and young cats under 56 weeks of age as high as 90% [22]. These studies examined either cats entering a hospital, or cats that were newly relinquished to a shelter, so a mixed population (consisting of cats originating from single- and multi-cat environments) can be assumed. This could explain the lower prevalence among adult cats when compared to the results of the present study.

Prevalence is expected to be higher when evaluating a population of cats from multi-cat households only. A few studies investigating FCoV shedding in a single cattery or shelter reported prevalence ranging from 73.8% to 100% [11,14,17], but these are not comparable to the present study, that investigated the prevalence of FCoV shedding in a population of cats from 37 different breeding catteries. Studies investigating FCoV antibody prevalence in catteries in the United Kingdom and in California, USA, found antibody prevalence of 84% [35] and 87% [36], respectively, but this is not comparable either, because antibody presence does not equal shedding.

Every cattery examined in the present study had at least one cat that was shedding FCoV; no cattery was free of the infection. There are several possible reasons. First, as shown in previous studies, multi-cat environments facilitate the spread of this highly contagious virus [9,29,37,38,39,40,41], and the fecal–oral route of transmission results in very effective propagation of FCoV through shared litter boxes, which is common practice in catteries [5,7,9,11,31,32,42,43]. After natural infection, cats start to shed high amounts of virus within seven days and continue to do so for several weeks or up to 18 months [7,9,43,44]. In most cats, shedding will gradually decrease after this initial phase and can even stop entirely, but cats remain susceptible to reinfection and will then start shedding again [7,11,22,32,43,44]. Some cats become lifelong shedders and only very few cats seem to be resistant and never shed the virus [7,11,22,29,32,43,44,45]. Second, catteries are usually home to kittens, which are known to shed the virus in particularly high amounts [5,22,32,43], and third, most cats in catteries are purebreds, which are discussed to be more susceptible to the infection [37,38].

According to previous studies, 70–80% of infected cats will become intermittent shedders [7,22,44,45]. Thus, these shedders could be missed when only a single fecal sample is analyzed [7,8,43,44]. Intermittent shedding can be caused either by reinfection or by intermittent virus excretion in persistently infected cats, and in both cases multiple phases without virus shedding can occur [7,8,43,44,46]. In order not to miss intermittent shedders, four samples from each cat (collected every 5–28 days) were analyzed in the present study. The proportion of shedding cats identified was significantly higher when all four samples of each cat were taken into account compared to only one sample per cat (76.5% and 61.5%, respectively; Table 2). These results support the recommendation that, for identification of FCoV shedders in a given population, serial fecal RT-qPCR tests should be performed [7,8,9,30,31,32].

The most suitable time interval for serial fecal sampling of an individual cat has not been clearly defined and the recommendations made by different authors vary from a few days to one month [7,8,30,31,32,39]. In the present study, no significant difference could be found when comparing separately calculated prevalence for different sampling intervals (5–9, 5–14, 5–21, or 5–28 days; Table 3). Thus, for detection of FCoV shedders, sampling intervals of one week to one month can be recommended.

Univariate risk factor analysis in the present study suggested that breed and the number of cats in the household had a significant influence on the prevalence of FCoV shedding, but these results have been distorted by the effect each breeder has on hygienic conditions and the risk of infection within their cattery. Additionally, most breeders keep only one, rarely two breeds within their cattery, so that the influence of the breed cannot easily be separated from the influence of the breeder. Multivariate risk factor analysis (GLMM) found no association between breed and FCoV shedding, suggesting that in this population, the breeders and their specific husbandry routine and hygiene management were truly influencing the prevalence of FCoV shedding, not the breed. However, if analysis had been performed with a greater number of cats from each breed, a significant influence of breed on FCoV shedding might have been found. Moreover, multivariate analysis revealed that the age of the cats was significantly associated with FCoV shedding in this population, while univariate analysis did not find a significant association between age and FCoV shedding. Thus, when considering the breeders’ effect, the influence of age on shedding becomes obvious.

The higher risk of FCoV shedding in young cats is consistent with previous studies [22,37,43]. Moreover, it was shown that kittens (under six months of age) also shed significantly more virus as determined by RT-qPCR than older cats [47]. Kittens in multi-cat households will usually acquire infection between the 6th and 10th week of age, when maternal antibodies wane [5,9,29,31,43]. Most previous studies could not demonstrate virus shedding before nine weeks [5,9,31,43], while Harpold and others demonstrated FCoV infection of kittens as early as four weeks of age [48]. Virus shedding starts a few days after the primary infection and is especially high and consistent in this early phase [5,43,46]. The higher frequency of shedding in kittens is likely due to the fact that the immune system is not fully developed and allows the virus to replicate efficiently [5,22,31,43].

The number of cats living together in one household was not significantly associated with FCoV shedding, once the breeders’ effect was controlled for. Living in a multi-cat household has already been confirmed as a risk factor for FCoV infection [5,8,29,35,36,37,49] and every cat tested in the present study came from a household with five or more cats. It can be concluded that for households with more than five cats, additional cats do not additionally increase the risk of FCoV shedding.

Hygiene management could have been expected to play a role in the distribution of FCoV, as the virus is transmitted via the fecal-oral route. In the present population, however, no significant association of hygiene management and FCoV shedding could be shown. Neither the number of litter boxes nor the cleaning and disinfection frequencies were associated with the prevalence of FCoV shedding. There are several possible explanations for this. First, the individual effect of the breeder on certain management-related risk factors was not assessed in the questionnaire, e.g., thoroughness of cleaning or the use of different cleaning agents. Second, it has been shown in previous studies that keeping cat populations with more than five cats free of FCoV is extremely difficult, due to the ubiquitous nature of the virus and the ease of transmission [3,31,43]. In order to prevent endless reinfection within such a cat population, shedders must be isolated, and the level of quarantine required to prevent contamination is extremely high and costly [5,31]. It is possible that hygiene-related factors, such as frequency of litter box cleaning and disinfection, do not have an influence on FCoV prevalence in households with more than five cats, because the virus is distributed so efficiently that normal sanitary measures are simply not enough to slow the spread.

Outdoor access has been suggested to reduce the risk of FCoV infection, because cats with outdoor access have the opportunity to bury their feces in distinct places and therefore have little to no contact with contaminated feces from other cats [8,22,31,49,50]. The results of previous studies are inconsistent regarding this topic. In an Australian study, cats living exclusively indoors had a higher prevalence of FCoV antibodies than cats with outdoor access, but the difference was not significant [49]. In a British study, feral and semi-feral cats were 3-times as likely as tame cats to have FCoV antibodies [51] and in another British study investigating risk factors for the presence of FCoV antibodies, feral cats did not have a reduced risk of having FCoV antibodies [37]. In the present study, there was no significant difference in FCoV shedding between cats with outdoor access and cats living indoors only. However, outdoor access in the present population did not refer to free-roaming, but to access to open-air enclosures on the breeders’ properties. The latter obviously did not differ much from keeping cats strictly indoors, because the cats will still use litter boxes or share places for defecation within the open-air enclosures.

Stress has been suggested to increase the risk of FCoV shedding [43] and the development of FIP [52]. A stress-related increase in glucocorticoid release is believed to be responsible for the suppression of cell-mediated immunity, resulting in higher FCoV replication [8,52]. One study could show an association between immunosuppression and an increased risk of FIP in feline immunodeficiency virus (FIV)-positive cats [53]. Another study examined the effect of stress on FCoV shedding in 29 experimentally infected cats by administering methylprednisolone acetate to ten of these cats. Seven additional cats became pregnant and had kittens during the experiment. No increase in virus shedding could be found in any of these cats, indicating that stress had no influence on FCoV shedding in the examined population [43]. Although a shelter environment is assumed to be more stressful for cats than a breeding cattery environment, the prevalence of FCoV shedding in this population was higher than many of the prevalence previously reported from shelters [16,20,23,54]. Thus, also a breeding cattery environment with more than five cats might represent a stressful situation for the individual animal.

This study had some limitations. First, the breeders’ statements in the epidemiological surveys were subjective and could not be verified. Second, not every cat from every participating cattery was tested, which impedes a direct comparison of catteries concerning management-related risk factors. Another limitation is that we cannot exclude that owners were inclined to specifically sample cats that were not healthy. However, as owners did not receive the results of this study immediately, there was no benefit of specifically picking those cats with disease signs. The limited number of cats from each breed is also a limitation which should be improved in future studies. Nevertheless, the fact that no cattery was FCoV-free confirms once more that large groups of cats, especially in breeding catteries, are at a high risk for FCoV infection. Additionally, keeping a large number of cats increases the risk of infection in such a manner that normal hygiene measures will not prevent the spread of the infection. Prevention strategies for FCoV infection and in general for virus infections are less likely to be successful when large groups of cats are kept together; smaller groups of cats should be preferred. The development of FIP currently can neither be predicted nor prevented once a cat is infected with FCoV, so the main focus should rather be on prevention of FCoV infection to avoid the development of this severe disease.

## 5. Conclusions

The overall prevalence of FCoV shedding in 37 German catteries was 76.5% (95% CI 69.8–82.2), and no cattery was identified to be free of FCoV. For identification of FCoV shedders in a multi-cat environment, at least three samples collected at intervals between one week and one month should be analyzed. In this population of cats from private multi-cat households housing more than five cats, only age was significantly associated with the risk of FCoV shedding. Young cats of less than one year of age had a 2.5-times higher risk of shedding FCoV than older cats. Hygiene management and limited outdoor access (with access to litter boxes) were not associated with FCoV shedding in this population.

## Figures and Tables

**Table 1:** Catteries and their prevalence of feline coronavirus (FCoV) shedding: Catteries are ranked in descending order by their prevalence of FCoV shedding. Prevalence was calculated as the number of cats positive for FCoV in at least one of the four samples (positive in 1/4, 2/4, 3/4, or 4/4 samples) divided by the number of cats tested. The numbers of cats positive for FCoV in 1/4, 2/4, 3/4, and all four samples are shown for each cattery.

Cattery Number	Number of Tested Cats Positive in at Least One Sample and Prevalence (%)	Number of Cats Tested Per Cattery	Total Number of Cats Living in Cattery	Number of Tested Cats Per Cattery Negative in All Samples	Number of Tested Cats Per Cattery Positive in 1/4 Samples	Number of Tested Cats Per Cattery Positive in 2/4 Samples	Number of Tested Cats Per Cattery Positive in 3/4 Samples	Number of Tested Cats Per Cattery Positive in 4/4 Samples	Breed(s) in Each Cattery
1	1 (100.0)	1	5	0	0	1	0	0	British Shorthair
2	5 (100.0)	5	15	0	0	0	0	5	British Shorthair
3	3 (100.0)	3	7	0	0	0	0	3	Turkish Angora
4	3 (100.0)	3	12	0	0	0	0	3	Maine Coon
5	2 (100.0)	2	10	0	0	0	1	1	Somali
6	7 (100.0)	7	15	0	0	0	0	7	British Shorthair
7	4 (100.0)	4	15	0	0	1	2	1	Maine Coon/Turkish Van
8	1 (100.0)	1	8	0	1	0	0	0	Scottish Straight
9	1 (100.0)	1	12	0	0	0	0	1	Birman
10	1 (100.0)	1	5	0	0	0	0	1	British Shorthair
11	1 (100.0)	1	9	0	1	0	0	0	British Shorthair
12	1 (100.0)	1	27	0	0	1	0	0	Oriental
13	3 (100.0)	3	7	0	1	0	0	2	Somali
14	2 (100.0)	2	6	0	0	0	0	2	Maine Coon
15	12 (100.0)	12	16	0	0	0	1	11	Persian
16	5 (100.0)	5	22	0	0	1	0	4	Birman
17	1 (100.0)	1	16	0	0	0	0	1	Persian
18	3 (100.0)	3	5	0	0	0	0	3	Scottish Fold/British Shorthair
19	4 (100.0)	4	14	0	1	1	0	2	Bengal
20	3 (100.0)	3	29	0	1	1	0	1	Bengal
21	9 (90.0)	10	16	1	1	2	0	6	British Shorthair
22	16 (88.9)	18	23	2	2	1	0	13	British Shorthair
23	13 (81.3)	16	20	3	3	1	0	9	Bengal/Sphynx/Taiga
24	4 (80.0)	5	12	1	0	1	0	3	British Shorthair
25	6 (75.0)	8	13	2	2	2	2	0	Bengal/Taiga
26	3 (75.0)	4	11	1	0	0	0	3	Bengal
27	3 (60.0)	5	5	2	0	0	0	3	British Shorthair
28	4 (57.1)	7	8	3	3	0	0	1	Norwegian Forest Cat
29	4 (57.1)	7	7	3	2	1	1	0	Bengal
30	1 (50.0)	2	15	1	0	1	0	0	Maine Coon
31	2 (50.0)	4	7	2	2	0	0	0	Maine Coon
32	1 (50.0)	2	5	1	0	0	1	0	Somali
33	1 (50.0)	2	28	1	0	0	0	1	Birman
34	2 (40.0)	5	15	3	1	0	0	1	Bengal
35	2 (33.3)	6	8	4	1	0	1	0	Norwegian Forest Cat
36	2 (28.6)	7	20	5	1	0	0	1	Norwegian Forest Cat
37	1 (12.5)	8	20	7	0	0	0	1	Birman
**Total**	**137 (76.5)**	**179**		**42**	**23**	**15**	**9**	**90**	

**Table 2:** Prevalence of feline coronavirus (FCoV) shedding for each cat was calculated separately by considering only the last collected sample (one sample per cat), the last two collected samples (two samples per cat), the last three collected samples (three samples per cat), and all four samples. Prevalence for two, three, and four samples per cat was then compared to the reference group (only one sample per cat) by using Fisher’s exact test.

Samples Per Cat	Number of Cats	Number of Cats Positive for FCoV in at Least One Sample	Prevalence (%)	CI (95%)	*p*-Value (Fisher’s Exact Test)
1	179	110	61.5	54.2–68.3	reference
2	179	123	68.7	61.6–75.1	0.1833
3	179	132	73.7	66.8–79.7	0.0175
4	179	137	76.5	69.8–82.2	0.0029

CI (95%) = 95% confidence interval.

**Table 3:** Cats were divided into four groups according to the longest of the time intervals between the collection of their four fecal samples: Group 1 included cats for which all time intervals were 5–9 days; Group 2 included cats for which the longest time interval was 10–14 days; Group 3 included cats for which the longest time interval was 15–21 days; Group 4 included cats for which the longest time interval was 22–28 days. Prevalence of feline coronavirus (FCoV) shedding was calculated separately for each group. A cat was considered a FCoV shedder when at least one of the four samples tested positive for FCoV. Groups 2, 3, and 4 were compared to the reference group 1 using Fisher’s exact test.

Maximum Number of Days Between Each Sample	Number of Cats	Number of Cats Positive for FCoV in at Least One Sample	Prevalence (%)	CI (95%)	*p*-Value
Group 1: 5–9 days	93	69	74.2	64.4–82.1	reference
Group 2: 5–14 days	42	36	85.7	71.8–93.7	0.1806
Group 3: 5–21 days	28	22	78.6	60.1–90.1	0.8040
Group 4: 5–28 days	16	10	62.5	38.5–81.6	0.3693

CI (95%) = 95% confidence interval.

**Table 4:** Evaluated categorical risk factors and their influence on feline coronavirus (FCoV) shedding in univariate and multivariate analyses. Fisher’s exact test was used for univariate analysis. A generalized linear mixed model (GLMM) was used for multivariate analysis.

Analyzed Risk Factor	Possible Categories/Values	Cats Tested (*n*)	Cats Positive for FCoV in at Least One Sample (%)	Univariate Analysis (*p*-Value)	GLMM		
					*p*-Value	OR	CI (95%)
age (categorized)	<1 year (a)	52	44 (84.6)	0.271	reference	reference	reference
	1–5 years (b)	104	76 (73.1)		0.042	2.48	1.03–5.95
	≥5 (c)	23	17 (73.9)		0.611	1.52	0.30–7.71
sex	female intact	107	81 (75.7)	0.419	eliminated by variable selection process		
	female neutered	11	7 (63.6)				
	male intact	48	37 (77.1)				
	male neutered	13	12 (92.3)				
breed	British Shorthair	54	48 (88.9)	<0.001	eliminated by variable selection process		
	Bengal	42	31 (73.8)				
	Norwegian Forest Cat	20	8 (40.0)				
	Birman	16	8 (50.0)				
	Persian	13	13 (100.0)				
	Maine Coon	12	9 (75.0)				
	Somali	7	6 (85.7)				
	Scottish Fold	2	2 (100.0)				
	Sphynx	3	3 (100.0)				
	Turkish Angora	3	3 (100.0)				
	Turkish Van	3	3 (100.0)				
	Taiga	2	1 (50.0)				
	Oriental	1	1 (100.0)				
	Scottish Straight	1	1 (100.0)				
number of cats in household	5–10 cats	53	35 (66.0)	0.036	eliminated by variable selection process		
	>10 cats	126	102 (81.0)				
frequency of litter box cleaning per day	once	52	37 (71.2)	0.463	eliminated by variable selection process		
	twice	85	68 (80.0)				
	3 times	19	14 (73.7)				
	4 times	0	0				
	≥5 times	23	18 (78.3)				
frequency of litter box disinfection per month	once or less	41	28 (68.3)	0.513	eliminated by variable selection process		
	more than once	138	109 (79.0)				
outdoor access	only indoors/balcony	92	74 (80.4)	0.221	eliminated by variable selection process		
	open-air enclosure	87	63 (72.4)				
contact to cats from other households	yes	46	39 (84.8)	0.658	eliminated by variable selection process		
	no	133	98 (73.7)				
feeding of raw meat	yes	43	32 (74.4)	0.108	eliminated by variable selection process		
	no	136	105 (77.2)				

CI (95%) = 95% confidence interval OR = Odds Ratio vs = versus.

**Table 5:** Evaluated continuous risk factors and their influence on feline coronavirus (FCoV) shedding in univariate and multivariate analyses. Mann–Whitney U test was used for univariate analysis. A generalized linear mixed model (GLMM) was used for multivariate analysis.

Analyzed Risk Factor	Group of CATS (n)	Median	IQR	Univariate Analysis *p*-Value	GLMM *p*-Value
ratio of cats and litter boxes (cats/litter boxes)	FCoV-positive (137)	2	1.1–2.2	1	eliminated by variable selection process
	FCoV-negative (42)	1.7	1.1–2.5		
available space in total (m^2^)	FCoV-positive (137)	165	110–200	0.748	eliminated by variable selection process
	FCoV-negative (42)	150	120–350		
available space per cat (m^2^)	FCoV-positive (137)	11	7–16	0.189	eliminated by variable selection process
	FCoV-negative (42)	14	8–20		

IQR = interquartile range.

## Supplementary Materials

The following materials are available online at https://www.mdpi.com/1999-4915/12/9/1000/s1: questionnaire for cat breeders used to assess signalment and anamnestic data (translated English version and original German version).
