# Supplementary material for: Prevalence of Feline Coronavirus Shedding in German Catteries and Associated Risk Factors
Source: Viruses. 2020 Sep 8;12(9):1000. doi: 10.3390/v12091000 (PMC7551668; doi:10.3390/v12091000)
Supplement: Supplementary file 1 [file viruses-12-01000-s001.zip › Prevalence of feline coronavirus shedding in catteries and associated risk factors - Questionnaire for cat owners - German version.pdf]

Fragebogen zur Untersuchung der Prävalenz und Korrelation mit Durchfall von felinen Coronaviren in Katzenzuchten

Datum ____________

Kotabsatz-Datum und Uhrzeit: ____

Besitzerinformationen

Name ____
Telefonnummer ____
Emailadresse ____
Postleitzahl und Ort ____

Informationen zur Katze

Name der Katze ____

Geburtsdatum oder ungefähres Alter der Katze ______________________________

Geschlecht männlich ☐ weiblich ☐

Diese Katze ist intakt ☐ kastriert ☐

Ist die Katze gegen FIP geimpft? ja ☐ nein ☐

Faecal score der Katze (vom <u>Untersucher</u> auszufüllen) 1 2 3 4 5 6 7

Informationen zur Haltung und Pflege der Katze

1. Fütterung: kommerzielles Futter ☐ selbstgekochtes Futter ☐
   Rohfütterung ☐
2. Trinkwasserquelle: Wasserhahn ☐ Toilette ☐ Vogelbad ☐
   Bach / Gartenteich / Pool ☐
   ausschließlich Mineralwasser ☐ Sonstiges: ________
3. Meine Katze lebt: nur im Haus ☐ als Freigänger ☐
   vor allem im Haus, aber mit eingeschränktem Freigang auf dem Balkon ☐ bzw. im Garten ☐
4. Gesamtzahl aktuell im Haushalt lebender Katzen ______________________
5. Alter und Anzahl aller im Haushalt lebenden Katzen:
   Anzahl Welpen bis 3 Monate ________ Anzahl Jungkatzen bis 1 Jahr ________
   Anzahl adulter Katzen bis 5 Jahre________ Anzahl adulter Katzen >5 Jahre ________
6. Werden alle Katzen der Zucht zusammen gehalten, oder gibt es kleinere Gruppen?
   alle zusammen ☐ Gruppenhaltung zu jeweils _____ Katzen
7. Gibt es noch andere Haustiere in Ihrem Haushalt außer Katzen?
   ja ☐ nein ☐
   Falls ja, welche Tierarten und wie viele? ______________________________

Fragebogen zur Untersuchung der Prävalenz und Korrelation mit Durchfall von felinen Coronaviren in Katzenzuchten

8. Gesamtzahl der Katzentoiletten im Haushalt ____________________

9. Mit wie vielen Katzen teilt sich diese Katze die Katzentoiletten? ____________

10. Wie oft am Tag werden die Katzentoiletten gereinigt? ________________

11. Wie oft werden die Katzentoiletten gewaschen und desinfiziert? __________

12. Werden noch andere Hygienemaßnahmen durchgeführt? ja ☐ nein ☐
Falls ja, welche genau? ______________________________

13. Hatte Ihre Katze schon einmal direkten Kontakt zu anderen Katzen außerhalb Ihrer Zucht? ja ☐ nein ☐
Falls ja, welche Art von Kontakt (z. B. Freigänger, Zuchtzwecke, Ausstellung,…)
____

14. Wie viele Quadratmeter beträgt die Fläche Ihres Haushaltes, zu der Ihre Katzen Zugang haben? ______________________________

15. Sind andere Katzen Ihrer Zucht gegen FIP geimpft? ja ☐ nein ☐
Falls ja, wie viele? alle ☐ alle, zu denen diese Katze Kontakt hat ☐
nur einzelne, zu denen diese Katze Kontakt hat ☐

16. Wogegen werden die Katzen Ihrer Zucht ansonsten regelmäßig geimpft?
Katzenschnupfenkomplex ☐ Feline Panleukopenie ☐
FeLV ☐ Tollwut ☐ sonstige Impfungen ☐

17. Wie häufig nehmen Sie neue Katzen in Ihren Bestand auf?
____

Krankheitsgeschichte

1. Gibt es irgendwelche bekannten chronischen Erkrankungen bei dieser Katze (Schilddrüsenüberfunktion, Nieren- oder Lebererkrankungen, Futtermittelallergien, o.ä.)?
____

2. Hatte diese Katze innerhalb der letzten 6 Monate weichen Kot oder Durchfall?
ja ☐ nein ☐
Falls ja,
wann und für wie lange? ______________________________
hat die Katze aktuell noch Durchfall? ja ☐ nein ☐
wie oft am Tag setzt/setzte Ihre Katze Durchfall ab?
1x ☐ 2x ☐ 3x ☐ 4x ☐ ≥5x ☐
war der Durchfall mit Gewichtsverlust verbunden? ja ☐ nein ☐
war dem Durchfall Schleim oder hellrotes Blut beigemengt)
ja ☐ ______________________________ nein ☐
wurde die Katze aufgrund dessen einem Tierarzt vorgestellt? ja ☐ nein ☐

wurde eine Diagnose gestellt? ja, ______________________ nein ☐
wurde der Durchfall mit Medikamenten behandelt? ja ☐ nein ☐
Falls ja,
welches Präparat? ______________________
war die Behandlung erfolgreich (Durchfall weg)? ja ☐ nein ☐

3. Wurde diese Katze in den letzten 6 Monaten auf gastrointestinale Parasiten untersucht? ja ☐ nein ☐
Falls ja, wie lautete die Diagnose?
Giardia spp. ☐ *Cryptosporidium spp.* ☐ Würmer ☐
Sonstiges ☐ ______________________

4. Wurde diese Katze in den letzten 6 Monaten gegen Parasiten behandelt (z. B. Wurmkuren)? ja ☐ nein ☐
Falls ja,
welches Präparat? ______________________
Dosierung und Dauer der Behandlung: ______________________
aus welchem Grund wurde behandelt?
routinemäßig ☐ andere Gründe ☐ ______________________

5. Hat diese Katze in den letzten 6 Monaten Antibiotika bekommen?
ja ☐ nein ☐
Falls ja,
welche Antibiotika wurden verabreicht? ______________________
wann und für wie lange wurde antibiotisch behandelt? ______________________
und warum wurde es angewendet? ______________________

6. Wurde bei dieser Katze jemals felines Coronavirus (FCoV) nachgewiesen?
ja ☐ nein ☐
Falls ja, mit welchem Verfahren?
FCoV-Antikörpernachweis aus dem Serum ☐
Virus-RNA-Nachweis per PCR aus dem Blut ☐ aus dem Kot ☐

7. Gab es im Leben dieser Katze ein oder mehrere besondere, mit Stress verbundene Ereignisse? (z. B. Umzug, Klinikaufenthalt, Geburt, Operation)
ja ______________________ (Beschreibung)
Falls ja, wann? ______________________
nein ☐

8. Hat diese Katze innerhalb der letzten 6 Monate immunsuppressive Medikamente erhalten? (z. B. Kortison/Glukokortikoide, Zytostatika)
ja, ______________________ (Präparat)
Falls ja, wann und für wie lange? ______________________
nein ☐

Fragebogen zur Untersuchung der Prävalenz und Korrelation mit Durchfall von felinen Coronaviren in Katzenzuchten

9. Hatten andere Katzen in Ihrem Haushalt in den letzten 6 Monaten Durchfall?
   ja ☐ nein ☐
   Falls ja, wie viele Katzen?______________________________

10. Wurden noch andere Katzen in Ihrem Haushalt in den letzten 6 Monaten auf gastro-intestinale Parasiten untersucht? ja ☐ nein ☐
    Falls ja, wie lautete die Diagnose? *Giardia sp.* ☐ *Cryptosporidium spp.* ☐
    Würmer ☐ Sonstiges __________________________

11. Wurde jemals bei einer anderen Katze Ihres Haushalts eine Infektion mit dem felinem Coronavirus (FCoV) festgestellt? ja ☐ nein ☐
    Falls ja,
    bei wie vielen und wann? __________________________
    welche Untersuchungen wurden gemacht?
    FCoV-Antikörpernachweis aus dem Serum ☐
    Virus-RNA-Nachweis per PCR aus dem Blut ☐ aus dem Kot ☐

12. Wurde jemals bei einer Katze Ihrer Zucht FIP diagnostiziert? ja ☐ nein ☐
    Falls ja,
    bei wie vielen Katzen? __________________________
    wann war das? __________________________
    wie wurde die Diagnose gestellt?
    Verdacht auf FIP anhand des klinischen Bildes ☐
    Verdacht auf FIP anhand von Veränderungen im Blut / Serum ☐
    mutierte Virus-RNA-Nachweis per PCR aus dem Blut ☐
    mutierte Virus-RNA-Nachweis per PCR aus dem Kot ☐
    Antikörper-Nachweis im Serum („FIP-Test“) ☐
    Titer: ________
    Untersuchung von Ergussflüssigkeit (Rivaltaprobe) ☐
    Nachweis von FCoV in Makrophagen im Erguss
    per Immunfloureszenz ☐
    oder in Gewebeproben per Immunhistochemie ☐
    pathologischer Nachweis von Organveränderungen ☐
