# Supplementary material for: Prevalence of Feline Coronavirus Shedding in German Catteries and Associated Risk Factors
Source: Viruses. 2020 Sep 8;12(9):1000. doi: 10.3390/v12091000 (PMC7551668; doi:10.3390/v12091000)
Supplement: Supplementary file 1 [file viruses-12-01000-s001.zip › Prevalence of feline coronavirus shedding in catteries and associated risk factors - Questionnaire for cat owners.pdf]

Survey for the investigation of prevalence of feline coronavirus shedding in catteries

Date ____________

Date and time of defecation: ______________________________

Owner´s information

Name ______________________________
Phone number ______________________________
E-mail address ______________________________
Postal code and city ______________________________

Information of cat

Name ______________________________

Date of birth or approximate age ______________________________

Sex male ☐ female ☐

This cat is intact ☐ neutered ☐

Has this cat been vaccinated against feline infectious peritonitis (FIP?) yes ☐ no ☐

Faecal score (to be answered by the investigator) 1 2 3 4 5 6 7

Information on housing and husbandry

1. Food: commercial diet ☐ home-cooked diet ☐
   raw diet ☐
2. Water source: tap water ☐ toilet ☐ bird bath ☐
   pond/creek/pool ☐ exclusively bottled water ☐
   other: ____________
3. Living conditions: only inside the house ☐ free-roaming ☐
   mainly inside, but access to balcony ☐ or secured garden area ☐
4. Total number of cats living in the household at this time ____________
5. Age and number of all cats living in the household:
   number of kittens <3 months ______ number of juveniles <1 year ______
   number of adults of 1-5 years ______ number of adults >5 years ______
6. Do all cats live together or are they separated into groups?
   all together ☐ separation into groups of ____ cats
7. Are there other pets in your household besides cats?
   yes ☐ no ☐
   If yes, what types and numbers of pets? ______________________________

8. Total number of litter boxes ______
9. With how many cats does **this** cat share its litter boxes? ______
10. How often do you clean the litter boxes per day? ______
11. How often do you wash and disinfect the litter boxes? ______
12. Do you apply any other hygiene measures? yes ☐ no ☐
    If yes, what exactly? ______
13. Did your cat ever have contact to cats from other households? yes ☐ no ☐
    If yes, what kind of contact (e.g. while free-roaming, for breeding purposes, at cat shows etc.) ______
14. Approximate size of your cat´s permanent living space (in m^2)? ______
15. Are other cats of your cattery vaccinated against FIP? yes ☐ no ☐
    If yes, how many? all ☐ all that live together with this cat ☐
    some that live together with this cat ☐
16. Against which infectious agents are your cats vaccinated routinely?
    Herpes-/Calicivirus ☐ Panleukopenia ☐
    FeLV ☐ Rabies ☐ other ☐
17. How often do new cats move into your household?

    ______

Medical history

1. Does this cat have any chronic diseases? (e.g. hyperthyroidism, kidney or liver disease, food allergies etc.)?

   ______

2. Did this cat have softer stool or diarrhoea within the last 6 months?
   yes ☐ no ☐
   If yes,
   when and how long? ______
   is diarrhoea still present now? ☐ yes no ☐
   how often (per day) did this cat have to defecate when having diarrhoea?
   1x ☐ 2x ☐ 3x ☐ 4x ☐ ≥5x ☐
   did this cat also lose weight? yes ☐ no ☐
   did the diarrhoea also contain blood or mucus?
   yes ☐ (please specify) ______ no ☐
   did you present the cat to a veterinarian for treatment? yes ☐ no ☐
   was a diagnosis established? yes, ______ no ☐
   was the diarrhoea treated medically? yes ☐ no ☐

If yes,

name of the medication: ____________________

was the treatment successful? yes ☐ no ☐

3. Has this cat been examined for intestinal parasites by faecal exam within the last 6 months? yes ☐ no ☐

   If yes, what has been found?

   Giardia spp. ☐ *Cryptosporidium spp.* ☐ *Strongyloides spp.* ☐

   other ☐ ____________________

4. Has this cat been treated (dewormed) for intestinal parasites within the last 6 months? yes ☐ no ☐

   If yes,

   which preparation? ____________________

   dosage and treatment period: ____________________

   reason for treatment: prophylactic treatment ☐

   other ☐ (please specify) ____________________

5. Did this cat receive antibiotics within the last 6 months?

   yes ☐ no ☐

   If yes,

   which preparation? ____________________

   dosage and treatment period: ____________________

   reason for treatment: ____________________

6. Has this cat ever been tested positive for feline coronavirus (FCoV)?

   yes ☐ no ☐

   If yes, what kind of test has been used?

   test for FCoV antibodies in the blood ☐

   test for viral RNA in the blood ☐ in the faeces ☐

7. Has this cat ever been exposed to special, stressful events? (e.g. relocation, hospitalization, parturition, surgery)

   yes ____________________ (please specify)

   when? ____________________

   no ☐

8. Did this cat receive any kind of immunosuppressive medication within the last 6 months? (e.g. prednisolone/other glucocorticoids, cytostatic agents)

   yes ____________________ (please specify)

   when and for how long? ____________________

   no ☐

9. Did other cats in your household have diarrhoea within the last 6 months?

   yes ☐ no ☐

If yes, how many cats? ______________________________

10. Have other cats of your household been examined for intestinal parasites within the last 6 months? yes ☐ no ☐

If yes, what has been found? *Giardia spp.* ☐ *Cryptosporidium spp.* ☐ *Strongyloides spp.* ☐ other ______________________________

11. Has any cat of your household ever been tested positive for FCoV?

yes ☐ no ☐

If yes,

how many and when? ______________________________

what kind of tests have been used?

test for FCoV antibodies in the blood ☐

test for viral RNA in the blood ☐ in the faeces ☐

12. Have any of your cats ever been diagnosed with FIP? yes ☐ no ☐

If yes,

how many cats? ______________________________

when? ______________________________

how was FIP diagnosed?

suspected on the basis of clinical presentation ☐

suspected on the basis of laboratory abnormalities (bloodwork) ☐

detection of mutated viral RNA by PCR (using a blood sample) ☐

detection of mutated viral RNA by PCR (using a faecal sample) ☐

detection of FCoV antibodies (using a blood sample) ☐

Titre: ________

Rivalta´s test (on abdominal effusion) ☐

detection of FCoV antigen in macrophages

in effusion, by immunofluorescence ☐

in tissue samples, by immunohistochemistry ☐

histopathological examination of organ samples obtained in necropsy ☐
